# Influence of Biopsychosocial Factors on the Survival of the Elderly in Northeast Brazil—A Prospective Study

**DOI:** 10.1155/2010/127605

**Published:** 2010-09-01

**Authors:** Álvaro Campos Cavalcanti Maciel, Ricardo Oliveira Guerra

**Affiliations:** Programa de Mestrado em Fisioterapia, Universidade Federal do Rio Grande do Norte, rua Moises Gosson, 1442, Lagoa Nova, CEP 59056-060 Natal-RN, Brazil

## Abstract

*Background*. Identifying the risk factors peculiar to each population has a great relevance, because it enables health policy formulators to analyze information accurately and by doing so, define objectives and action programs aimed at a qualitative and economically feasible solution to the problem. Thus, this study aimed at identifying the risk factors for survival in elderly in a city in the state of Rio Grande do Norte (RN), Brazil. *Methods*. A prospective study was carried out, where 310 elderly persons were selected to form a baseline. The follow-up was 53 months. The predictive variables were divided into sociodemographic, physical health, neuropsychiatric and functional capacity. Statistical analysis was performed using bivariate analysis, survival analysis, followed by Cox regression in the multivariate analysis. *Results*. A total of 60 (19.3%) elderly individuals died during the follow-up. The survival mean was 24.8 months. In the Cox analysis, dependence in basic activities of daily living (HR = 3.55), cognitive deficit (HR = 4.22) and stroke (HR = 3.35) continued as independent risk factors for death. *Discussion*. The risk factors found in the study can be interpreted as the primary predictors for death among elderly members of the community.

## 1. Introduction

The aging process has had considerable influence on the development and functioning of societies, to such an extent that all countries tend to give priority to the possible repercussions of this progression in the areas of public health and national economy [1]. 

In Brazil, the rapid growth of the elderly population is making the demographic transition process much more complex than in other parts of the world. These sociodemographic changes have not been occurring homogeneously in all the regions or even in the cities of a same state [2, 3].

This situation results in important differences and a wide diversity in the health levels of the elderly, owing to socioeconomic factors. Several studies have shown that the result of the interaction between these factors and physical and mental health as well as environmental aspects influences the quality of life of the elderly and they are fundamental factors for the morbidity and mortality indices [4–6].

When considering the elderly as belonging to the most vulnerable groups, where the various risk factors are interconnected, we observe the need for special attention from the health care point of view. However, for this to occur efficiently, we must know the situation in each region and thereby identify the conditions that predispose the elderly to a greater risk of developing the event of interest [7].

Of the few longitudinal studies performed in Brazil, the main risk factors found for mortality are age itself and sex, where the risk for men is greater than that for women [8, 9]. All the remaining variables are dependent on a complex interaction between the individual and the environment, which varies from culture to culture and over time. Recent years have seen an increase in studies on risk factors and variables such as cognitive function and perceived health, to such an extent that, there is agreement in the current literature that they are strong predictors of death over time [10, 11].

Identifying the risk factors peculiar to each population has economic and sanitary relevance, because it enables health policy formulators to analyze information accurately and by doing so, define objectives and action programs aimed at a qualitative and economically feasible solution to the problem [7, 8]. 

Given the scarcity of prospective studies on the elderly Brazilian population, mainly in the rural zones, the aim of this study is to identify the risk factors for death in relation to the sociodemographic aspects, physical and mental health, and functional capacity, in a cohort study of elderly individuals in a rural city in the state of Rio Grande do Norte (RN) Brazil.

## 2. Methodology

### 2.1. Study Design

This is a prospective, longitudinal study, with a 53-month followup.

### 2.2. Population and Sample

The population consisted of 3070 elderly persons, aged 60 years or more, inhabitants in the urban zone of Santa Cruz, Brazil and enrolled in the Basic Care Information System (SIAB), in December 2001.

To calculate the sample at baseline, we adopted the following statistical parameters: maximum statistical errors of 5% for type I and 20.0% for type II errors, with a power of 80% for the study. For a deviation of ±5% in the estimates made and considering the reference population of 3070 elderly over the age of 60 years, and residents of Santa Cruz in 2001, a sample of 282 individuals was determined. A total of 28 individuals (10%) were added to this number to compensate for possible losses during the followup.

Thus, 310 persons were selected in a systematic random probabilistic manner to form the baseline. Considering the heterogeneity of the location and to obtain a representative sample, the city was divided into three socioeconomically different zones from the five existing districts, where Paraiso, Conjunto Conego Monte, and Centro have the worst, intermediate, and best socioeconomic level, respectively.

Baseline data collection was carried out between August and October 2002 and the reassessment between January and March 2007, considering the 53 months of the study. The interviews took place in the homes of the elderly. Mortality information was obtained from death certificates supplied by the family, or from the municipal registry office. The interviews were conducted by the same individual, duly trained in a previous pilot study, and the same person who interviewed the elderly at the beginning of the research.

After 53 months, 293 individuals were located (94.5% of the initial sample), of which 233 (75.2%) were reassessed, 60 (19.3%) had died, 17 (5.5%) had dropped out, because the death certificate could not be found, or that person's address.

### 2.3. Description of the Study Site

The city, containing around 32,000 inhabitants, is located 120 km from Natal (capital of the state of Rio Grande do Norte), in the Trairi region. The study region is poor and underdeveloped, and this is reflected in the low socioeconomic conditions of most of the population. A majority of the residents are rural workers who suffer long periods of drought, owing to the variable rainfall. These characteristics of poverty are evident when one analyzes the regional development indicators, such as: Human development Index (HDI) of 0.65, per capita GDP of R$2,100.00 (≈US $900.00) and 25.49% illiteracy rate above the age of 15 years.

### 2.4. Variables

The predictor variables were allocated to four groups.

- Sociodemographic variables: age, sex, schooling, marital state, number of persons in the household, race, and leisure activities.
- Physical health variables: self-declared pathologies present at the interview [12] (Diabetes mellitus, cardiovascular disease, pulmonary disease, stroke, hip fracture, rheumatism, visual and auditory impairment and cancer), self-perception of health (satisfied or unsatisfied), number of medications in continual use, and number of hospitalizations in the last year.
- neuropsychiatric variables: the Geriatric Depression Scale (GDS-15)^13^ was used to assess the presence of depressive symptoms. The cutoff point adopted was that proposed by Paradela et al. [13]. Pfeiffer's Short Portable Mental Status Questionnaire (SPMSQ) [14] was used to evaluate cognitive function. The cutoff point adopted was proposed in the scale validation study for the Brazilian population [15].
- Functional capacity: the Katz Index [16] was used to assess functional capacity for the basic activities of daily living (BADLs). The index is composed of the following activities: bathing, dressing, toileting, climbing up/down stairs, and sphincter control. The cutoff point adopted was that proposed in the scale validation study for the Brazilian population [17].

The dependent variable was the occurrence of death, expressed in the bivariate analysis by vital status. From the information of the occurrence of the event death, were also collected: cause of death and time to death.

### 2.5. Statistical Analysis

Data processing, storage, and analysis were performed by the SPSS (Statistical Package for the Social Sciences) 15.0 program. Descriptive statistical analysis was carried out using distribution measures (mean, median, standard deviation, absolute frequency, and relative frequency). 

Bivariate analysis showed an association between the independent variables and the dependent variable vital status (whether the elderly individual was alive or dead), using contingency tables and comparing the results using Pearson's chi-square (*χ* ^2^) test with Yates' continuity correction. Similarly, survival curves were drawn using the Kaplan-Meier product-limit method, considering the initial limit as being the month in which the first interview took place, and the last, the reassessment month. The log-rank test was applied to compare between the curves obtained for different categories of the same variable.

Multivariate analysis was then performed using Cox regression to estimate the effect of predictor variables on the survival of elderly people. The exit criterion for all the variables introduced into the model was *P* < .10. At the end, a regression model was obtained containing only statistically significant variables, expressed by hazard ratios (HR). All the statistical analysis was done considering an *α* value of 0.05 and confidence interval (CI) of 95%.

### 2.6. Ethical Aspects

The present study involved the use of information obtained through interviews conducted in the homes of elderly individuals. After data collection, the records were kept by the researcher and at no time were the interviewees identified nor was their personal information made available to others.

The research protocol was approved by the Research Ethics Committee of the Universidade Federal do Rio Grande do Norte (protocol no. 84/02).

## 3. Results

Tables 1, 2, and 3 shows the sample characteristics at baseline by vital status. There were 60 deaths (19.3%), representing an annual rate of 4.8%, the main cause being cardiovascular disease (41.4%), followed by stroke.

The analysis between sociodemographic variables and the dependent variable vital status showed a significant association with death, age (*P* < .001), leisure activities (*P* < .001), and marital status (*P* = .001) (Table 1). With respect to physical health, a significant association was found with pulmonary disease (*P* = .002), hip fracture (*P* < .001), number of medications (*P* = .001), number of hospitalizations (*P* < .001), stroke (*P* = .001), auditory impairment (*P* = .001), and perception of health (*P* = .001) (Table 2). There was a significant association in all situations involving neuropsychiatric and functional capacity variables (Table 3).

**Figure 1:**
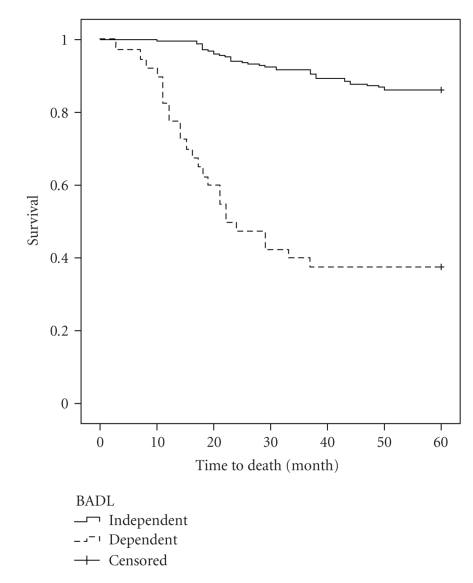
The Kaplan-Meier curve for ABDL according to the two groups, during the follow-up period.

**Figure 2:**
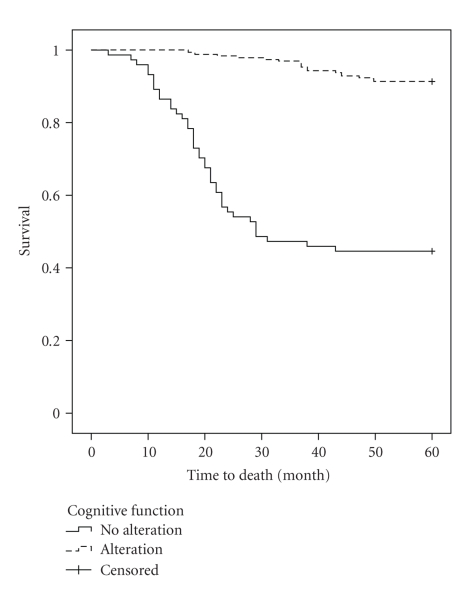
The Kaplan-Meier curve for cognitive function according to the two groups, during the follow-up period.

Survival was 24.8 months (SD = ±12), ranging from 3 to 50 months. The study of overall survival demonstrated survival rates of 97%, 54%, 31%, and 5% at 12, 24, 36, and 48 months of followup, respectively. These values indicate a higher incidence of deaths in the first year of followup (43%) and in subsequent years, a constant reduction of around 25%. Analysis of the survival curves for each predictor variable showed a significant difference in the curves found, only in the limitation for BADL (log-rank = 22.12; df = 1; *P* < .001) and in cognitive impairment (log-rank = 27.13; df = 1; *P* < .001), as illustrated in Figures 1 and 2, respectively.

In the Cox regression model, the independent risk factors, in terms of survival, in addition to dependence in BADL (HR = 3.55), were stroke (HR = 3.35) and cognitive impairment (HR = 4.22), as shown in Table 4.

## 4. Discussion

The aim of this study was to analyze the behavior of predictor variables during a longitudinal study, as risk factors for the survival of elderly residents in the community. The prospective followup of the elderly—unlike cross-sectional studies, where it is difficult to determine the direction of causality—serves to analyze the relationships between the sample characteristics at baseline and their subsequent alterations, and accurately estimate the magnitude of the influence of the variables on the outcome obtained.

With respect to the percentage of deaths that occurred during followup, the values found were quite similar to those obtained in other studies conducted in Europe [18–20]. In Brazil, two important studies with the same characteristics, carried out in the Southeast region, also describe comparable variables, ranging between 15% and 23%, mainly as a function of follow-up time [3, 21]. Considering the annual mortality rate for the Brazilian population (around 4.3% for those over 60 years of age), these values are close to the national average [22].

Among the causes of death, cardiovascular disease was the main pathology found on the death certificates. Diseases of this nature are generally considered important risk factors for incapacity and death. Despite diagnostic and treatment advances, the aggressive and sometimes insidious way in which they present themselves prevents therapeutic measures from reversing this picture and avoiding death [8].

Multivariate analysis by Cox regression showed that cognitive impairment, the limitation in BADLs, and stroke were important predictors of death in the population studied.

Indeed, cognitive impairment is currently a significant public health problem, with social and economic repercussions that affect both the elderly and their families. The presence of this condition, by itself, may be a factor for the emergence of other risk factors in the elderly, such as functional limitation, falls, hospitalization, and affective alterations such as depressive symptoms, anxiety disorders, and insomnia [19].

This situation is more chaotic in underprivileged areas because families do not benefit from any health support or social network. In a study performed in Taiwan, it was observed that social support networks for the elderly provided substantial protection against the emergence of cognitive alterations during followup. This protector effect of social support is even greater than that of family companionship [23]. Other studies carried out in Latin America and in Brazil show the relationship between mental problems (cognitive impairment, depression, and anxiety) as socioeconomic indicators [24–26].

Another aspect that must be considered in this analysis is the impact of cognitive dysfunction on the health system. The absence of social incentive policies for families often results in admission to nursing homes or extended hospitalization, in contrast to the tendencies of effective treatments that aim at keeping the elderly in the community by promoting integrated home care services and day centers [27].

The question of financial burden on the health system is also relevant when cognitive impairment is considered. In a study conducted in California, it was found that patients with Alzheimer's disease and at high risk of dying represent a monthly expense of more than 700 dollars, compared to those considered at low risk [28]. Thus, reducing the prevalence and impact of cognitive impairment, in addition to increasing life expectancy, may result in decreased health costs [29].

The second predictor variable was the limitation in the basic activities of daily living, derived from the analysis of functional capacity. Functional capacity is defined as the capacity of individuals to independently perform activities considered essential to their survival and, consequently to the preservation of their social relationships [30, 31]. Its study has become very useful in assessing the health status of this population.

From the public health viewpoint, functional capacity emerges as a new health concept, which is more appropriate for instrumentalizing and operationalizing health care for the elderly. Preventive, supportive, and rehabilitative measures must aim at improving functional capacity or, at least, at preserving it, and, whenever possible, at recovering the capacity lost by the elderly individual [32, 33]. Thus, incapacity is seen not as an attribute, but as a complex grouping of conditions, many of which are created by the social environment [1].

In this sense, the social environment of the city of Santa Cruz may negatively influence the functionality of individuals. As a result, social support ineffective increases relationship between aging and diseases, limitations in daily activities and permanent disability, and reduce the supply of health education programs [34]. 

Both cross-sectional and longitudinal studies have reported that high indices of social activity are associated with improved functionality. Taking part in community events, making or receiving visits, and attending religious services are cited as being favorable in preserving functional capacity [7, 8].

Considering specifically basic activities, the fact of needing help to perform simple daily tasks, such as eating, bathing, and walking, added to the inadequate medical structure for meeting elderly demands, is associated to a large number of negative health indicators, such as hospitalization, treatment costs, quality of life, and finally, death [31].

Another predictor of death was stroke. Cerebrovascular disease is the primary cause of incapacity and the third cause of death, surpassed only by cardiac diseases and cancer. Its worldwide incidence has been estimated at 300 cases for every 100,000 persons, with a slight predominance in men aged between 45 and 84 years. According to a study conducted in Brazil between 1980 and 1995, cerebrovascular diseases account for one third of the annual deaths from disorders of the circulatory system [35].

In general, recent years have shown a decline in death from stroke. This may be a consequence of the decline in lethality, due to improved treatment of the disease or of its complications, to less severe cases or because of the decreased incidence of new stroke cases. Another point to be considered is the influence of health care on the decline in death by stroke [36].

A number of authors have suggested that most deaths from stroke have occurred outside the health care system, and that the decline is related to environmental, social, or cultural factors and to health-related behavioral changes. Others, however, believe that the accelerated fall as of the 1970s is a result of more effective health care [35, 37]. Considering the reality of the local health system, where the offer of health services and prevention programs is scarce, it is plausible that stroke may indeed be an important predictor of death in this community.

An analysis of the risk factors found shows a close relationship between cognitive function, stroke, and functional capacity, in such a way that the possible associations observed increase substantially the risk of death in the elderly. The aging process is a result of the interaction between physical and mental health, functional independence, social integration, as well as family and economic support.

Compromised cognitive performance may alter functional capacity and jeopardize other domains, such as the physical and the social. Cognitive impairment and mainly dementia lead to loss of autonomy and independence and is associated to greater risk of death [38]. Similarly, there is a complex network of events that relate cognitive impairment with stroke. In a study carried out in the United Kingdom, an incidence between 23% and 45% of cognitive impairment was observed in subjects with stroke, varying as a function of the subject's age [39]. Conversely, a study performed to identify racial and geographic differences showed cognitive impairment associated with other variables, such as high blood pressure and diabetes, to be an important risk factor for stroke. The presence of alterations in temporal orientation and in language could be related to a likely vascular disorder, thus favoring the occurrence of stroke [37]. This being so, the presence of cognitive impairment, associated to functional limitations and stroke in the elderly of Santa Cruz, seems to have added another independent risk of death. We also examine the possibility of extrapolating our findings to regions with sociodemographic, cultural pattern, and lifestyle similar.

Thus, there is an obvious need to investigate how the different aspects involved in the daily life of the elderly influence their health and, consequently, their death. The adoption of explicative models of this process and the understanding of transition patterns help in designing studies, favoring the development of therapeutic intervention programs to combat incapacitating diseases, as well as in planning health care measures aimed at preventing incapacities and reducing the number of deaths.

## Figures and Tables

**Table 1:** Distribution of vital status, according to sociodemographic variables of elderly residents in Santa Cruz, Brazil.

Variables	Vital status				*P*
	Living		Dead		
	*n*	%	*n*	%	
Age					
60–75 years	148	90.2	16	9.8	<.001
Over 75 years	85	65.9	44	34.1	
Sex					
Male	83	79.0	22	21.0	.88
Female	150	79.8	38	20.2	
Race					
White	78	72.2	30	27.8	.06
Mixed	132	83.5	26	16.5	
Black	23	85.2	4	14.8	
Schooling					
Illiterate/semi-illiterate	88	75.2	29	24.8	.13
Literate	145	82.4	31	17.6	
Marital status					
Married/common law	140	86.4	22	13.6	.01
Single/widowed	93	71.0	38	29.0	
Leisure activities					
Yes	125	89.9	14	10.1	<.001
No	108	70.1	10	21.3	

**Table 2:** Distribution of vital status, according to physical health variables of elderly residents in Santa Cruz, Brazil.

Variables	Vital status				
	Living		Dead		*P*
	*n*	%	*n*	%	
Diabetes melitus					
Yes	35	76.1	11	23.9	.53
No	198	80.2	49	19.8	
Cardiovascular disease					
Yes	111	78.2	31	21.8	.57
No	122	80.8	29	19.2	
Stroke					
Yes	9	37.5	15	62.5	.001
No	224	83.3	45	16.7	
Pulmonary diseases					
Yes	18	58.1	13	41.9	.002
No	215	82.1	47	17.9	
Hip fracture					
Yes	4	33.3	8	66.7	<.001
No	229	81.5	52	18.5	
Rheumatism					
Yes	122	77.7	35	22.3	.40
No	111	81.6	25	18.4	
Visual impairment					
Yes	176	76.2	55	23.8	.06
No	57	91.9	5	8.1	
Auditory impairment					
Yes	83	72.2	32	27.8	.01
No	150	84.3	28	15.7	
Cancer					
Yes	6	66.7	3	33.3	.33
No	227	79.9	57	20.1	
Self-perception of health					
Satisfied	120	92.3	10	7.7	.001
Unsatisfied	97	73.5	35	26.5	
Number of medications					
None/one	179	84.4	33	15.6	.001
More than one	54	66.7	27	33.3	
No of hospitalizations (in the last year)					
None	222	88.2	48	17.8	.001
One or more	11	47.8	12	52.2	

**Table 3:** Distribution of vital status, according to neuropsychiatric and functional capacity variables of elderly residents in Santa Cruz, Brazil.

Variables	Vital status				
	Living		Dead		*P*
	*n*	%	*n*	%	
Cognitive function					
Altered	33	44.6	41	55.4	<.001
Unaltered	200	91.3	19	8.7	
Symptoms of depression					
Present	55	72.4	21	27.6	.005
Absent	176	86.7	27	13.3	
BADL					
Dependence	15	37.5	25	62.5	<.001
Independence	218	86.2	35	13.8	

**Table 4:** Result of the Cox Regression, with the predicting variables of mortality of elderly from Santa Cruz, Rio Grande do Norte, Brazil.

Variables	Model 01		Model 02		Model 03		Model 04	
	HR	IC 95%	HR	IC 95%	HR	IC 95%	HR	IC 95%
Female sex	1.56	0.79–2.22 *P* = .57	1.45	0.80–2.56 *P* = .21	1.40	0.76–2.35 *P* = .36	1.21	0.70–2.12 *P* = .47

Age (years)	1.05	1.01–1.10 *P* = .002	1.06	1.03–1.12 *P* = .02	1.11	1.01–1.24 *P* = .044	1.04	0.99–1.09 *P* = .11

No leisure activities			1.41	0.71–2.82 *P* = .32				

Marital status (single/widowed)			0.95	0.49–1.81 *P* = .88				

Hip fracture					0.53	0.16–1.72 *P* = .29		

Unsatisfied self-perception of health					1.36	0.47–3.90 *P* = .56		

Visual impairment					2.12	0.65–4.56 *P* = .56		

Auditory impairment					6.56	0.55–14.61 *P* = .65		

Stroke					3.99	2.13–5.56 *P* = .001	3.35	1.18–7.80 *P* = .002

No of hospitalizations (more than one)					0.59	0.20–1.45 *P* = .22		

Number of medications (more than one)					0.92	0.43–2.00 *P* = .85		

Cognitive alteration							4.22	2.01–8.97 *P* = .001

Symptoms of depression							1.09	0.58–2.02 *P* = .78

Dependence in BADL					3.68	1.95–6.77 *P* = .001	3.55	1.77–6.45 *P* = .001
